# Anticoagulation Intensity of Rivaroxaban for Stroke Patients at a Special Low Dosage in Japan

**DOI:** 10.1371/journal.pone.0113641

**Published:** 2014-11-26

**Authors:** Takuya Okata, Kazunori Toyoda, Akira Okamoto, Toshiyuki Miyata, Kazuyuki Nagatsuka, Kazuo Minematsu

**Affiliations:** 1 Department of Cerebrovascular Medicine, National Cerebral and Cardiovascular Center, Osaka, Japan; 2 Department of Clinical Chemistory, National Cerebral and Cardiovascular Center, Osaka, Japan; 3 Department of Molecular Pathogenesis, National Cerebral and Cardiovascular Center, Osaka, Japan; 4 Department of Neurology, National Cerebral and Cardiovascular Center, Osaka, Japan; Federico II University of Naples, Italy

## Abstract

**Objectives:**

In Japan, low-dose rivaroxaban [15 mg QD/10 mg QD for creatinine clearance of 30–49 mL/min] was approved for clinical use in NVAF patients partly because of its unique pharmacokinetics in Japanese subjects. The aim of the study was to determine the anticoagulation intensity of rivaroxaban and its determinant factors in Japanese stroke patients.

**Methods:**

Consecutive stroke patients with NVAF admitted between July 2012 and December 2013 were studied. Prothrombin time (PT), activated partial thromboplastin time (aPTT), and estimated plasma concentration of rivaroxaban (C_riv_) based on an anti-factor Xa chromogenic assay were measured just before and 4 and 9 h after administration at the steady state level of rivaroxaban. Determinant factors for C_riv_ were explored using a linear mixed-model approach.

**Results:**

Of 110 patients (37 women, 75±9 years old), 59 took 15 mg QD of rivaroxaban and 51 took 10 mg QD. C_riv_ at 4 h was 186 ng/mL for patients taking 15 mg QD and 147 ng/mL for those taking 10 mg QD. Both PT and aPTT were positively correlated with C_riv_. C_riv_ was 72% lower at 4 h in 15 patients receiving crushed tablets than in the other patients, and tablet crushing was significantly associated with lower C_riv_ (adjusted estimate −0.43, 95% CI −0.60 to −0.26) after multivariate-adjustment.

**Conclusion:**

The anticoagulation effects of rivaroxaban in the acute stroke setting for Japanese NVAF patients were relatively low as compared with those in the ROCKET-AF and J-ROCKET AF trials. Tablet crushing, common in dysphagic patients, decreased C_riv_.

## Introduction

Atrial fibrillation (AF) is associated with an increased risk of stroke and thromboembolism, and effective antithrombotic therapy significantly reduces this risk [1]. Oral anticoagulant therapy with vitamin K antagonists (VKAs) has been established as the standard for stroke prevention in patients with AF [2]. Recently, novel oral anticoagulants (NOACs) have emerged as an alternative to VKAs for thromboembolic prevention in patients with nonvalvular AF (NVAF). Among these, rivaroxaban (Bayer Schering Pharma AG, Wuppertal, Germany) is an oral direct activated coagulation factor X (FXa) inhibitor that binds directly to the catalytic site of the serine protease FXa independently of antithrombin and inhibits both free and prothrombinase-bound FXa [3].

To reduce the risk of stroke and systemic embolism in patients with NVAF, special low dosages of rivaroxaban are recommended in Japan; i.e. 15 mg quaque die (QD) for patients with creatinine clearance (CrCl) ≧50 mL/min, and 10 mg QD for those with CrCl of 15–49 mL/min, as compared to globally approved dosages of 20 mg QD and 15 mg QD, respectively. This recommendation was based on the unique pharmacokinetics in Japanese subjects showing higher rivaroxaban exposure than Caucasian subjects when using the same dosage [4], and the Japanese Rivaroxaban Once Daily Oral Direct Factor Xa Inhibition Compared with Vitamin K Antagonism for Prevention of Stroke and Embolism Trial in Atrial Fibrillation (J-ROCKET AF) proved the safety and efficacy of this low-dose rivaroxaban medication in Japanese NVAF patients [5]. However, the anticoagulation effect of rivaroxaban, especially with the low dosage, has been understudied in the acute stroke setting because acute stroke patients were excluded in the above pharmacokinetics study and trial. For example, the ROCKET-AF, J-ROCKET AF, RELY, and ENGAGE AF-TIMI 48 excluded acute stroke patients within 14 days after onset [5],[6],[7],[8], and the ARISTOTLE excluded those within 7 days from enrollment [9]. Thus, these major trials did not prove the efficacy and safety of NOACs for acute stroke patients at all. Various clinical conditions associated with stroke, such as highly advanced age, differences in drug administration, and potential damage to the kidney and other organs by acute stroke effects, might affect anticoagulation intensity. To examine the issue of the anticoagulation effect of rivaroxaban in clinical practice, the aim was to determine the anticoagulation intensity of rivaroxaban and its determinant factors in Japanese patients with stroke.

## Methods

### Ethic Statement

The study conformed to the guiding principles of the Declaration of Helsinki and was approved by the local ethics committee of National Cerebral and Cardiovascular Center. All patients or their next of kin gave their written informed consent to participate.

### Patients and demographic data

Among patients admitted to our cerebrovascular unit due to stroke and transient ischemic attack (TIA) from July 2012 through December 2013 (recruitment interrupted from August 2013 to November 2013 due to technical problems), data of patients who had NVAF and started to take rivaroxaban for the prevention of stroke and systemic embolism were collected prospectively.

The baseline characteristics of patients, including components of the CHADS_2_ and CHA_2_DS_2_-VASc scores, weight, National Institutes of Health Stroke Scale (NIHSS) score, renal function, and other medications on the day of blood collection, as well as whether the rivaroxaban tablet was crushed, were recorded. Renal function was expressed as CrCl using the Cockcroft and Gault equation.

### Blood Collection and Measurements of Coagulation Assays

All patients took rivaroxaban after breakfast. Blood sampling was performed at least 2 days after rivaroxaban was started, when its concentration was considered to have reached steady state. Two venous blood samples were collected each time in citrate-containing tubes just before (0 h) and 4 h and 9 h after drug administration. The sampling point at 4 h was meant to capture the maximum concentration of rivaroxaban because the maximum concentration has been reported to occur 1 to 3 h after tablet intake and to be delayed by 2 h with food [10]. The sampling point at 9 h was meant to reflect the half-life of rivaroxaban, which has been reported to be 11 to 13 h in the elderly, partly due to renal dysfunction [11]. For 1 of the 2 tubes, following double centrifugation at 2,500 g for 15 min, platelet-poor plasma was collected, quick-frozen, and stored at −80°C until the analysis for anti-FXa activity was performed. Blood samples were drawn into a citrate-containing tube using a 21-gauge needle. The prothrombin time (PT, Recombiplastin [Instrumentation Laboratory, Bedford, MA, USA] and activated partial thromboplastin time (aPTT, Actin [Siemens Healthcare Diagnostics Inc., Tarrytown, NY, USA]) were measured immediately, and the calibrated plasma rivaroxaban concentration (C_riv_) was analyzed based on the anti-FXa activity (anti-factor Xa chromogenic assay, STA-Liquid Anti-Xa [Diagnostica Stago, Asnières, France]) of the stored samples. Anti-factor Xa chromogenic assays have previously been shown to have acceptable accuracy and precision [12], and they have been recommended for quantitative measurements of rivaroxaban exposure, using rivaroxaban calibrators with results expressed as ng/mL of rivaroxaban. The minimum detectable sensitivity of estimated rivaroxaban concentration based on the anti-factor Xa chromogenic assay was 10 ng/mL. If the estimated rivaroxaban concentration was below the limit of detection, it was treated as 5 ng/mL for convenience. These assays were all measured on a STA-R coagulometer (Diagnostica Stago, Asnières, France).

### Statistical analysis

Data are presented as values and percentages, means ± SD, or medians (interquartile range). Rivaroxaban concentrations were log-transformed due to right skewness of the original distributions (log C_riv_). The baseline characteristics and laboratory profiles were compared by rivaroxaban dosage subgroup using the Wilcoxon signed-rank test for continuous variables and the chi-square test or Fisher’s exact test for categorical variables.

In order to identify variables affecting rivaroxaban concentrations at the three fixed time points, a linear mixed-effects (LME) model approach was adopted. LME models are statistical models that are used in the analysis of clustered or longitudinal data. LME models estimate the relationship between the dependent variable and the predictors included in the model, accounting for both the fixed effects and the random effects of the independent variables. Compared with linear regression models without considering clustering or temporal effects, LME models are able to more accurately estimate the fixed effects by estimating the covariance structure through the inclusion of individual-specific random effects [13]. First, for the purpose of selecting the variables to be included in the model, the effects of various baseline characteristics on rivaroxaban concentrations were evaluated, using a LME model with fixed effects for each variable and time points of blood sampling and a random effect for patients. Second, variables with P<0.20 and time points of blood sampling were included as fixed effects in the LME model performed with rivaroxaban concentrations, whereas patients were treated as a random effect. The level of significance was set at 95% (P = 0.05). Statistical analysis was performed using JMP, version 10.0.2 (SAS Institute Inc., Cary, NC, USA).

## Results

### Patients’ Characteristics

A total of 126 patients started to take rivaroxaban. Of these, seven patients who did not consent to participate, one with off-label dosage (7.5 mg QD), and eight who took rivaroxaban with the evening meal were excluded. Thus, 110 patients (37 women, 75±9 years old) were studied. All patients had breakfast or tube feeding in the morning on the day of blood collection. Fifty-nine patients (54%) took 15 mg QD of rivaroxaban, and the other 51 took 10 mg QD. Thirty-seven patients (34%) taking 10 mg QD had renal function of CrCl 30–49 mL/min. In addition, six patients with prior intracerebral hemorrhage, one with prior muscular hemorrhage, and seven very elderly patients took 10 mg QD based on the judgment of the physician in charge even though their CrCl values were 50 mL/min or greater. Eighty-four patients (76%) were hospitalized due to acute ischemic stroke, and rivaroxaban was initiated at a median of 5 days after symptom onset. Eight patients were hospitalized due to acute TIA (initiation of rivaroxaban at a median of 2.5 days), and seven were hospitalized due to acute intracerebral hemorrhage (at a median of 11 days). The other 11 patients were hospitalized due to chronic ischemic stroke. The baseline characteristics of the patients are shown in Table 1. Fifteen patients received crushed rivaroxaban tablets due to dysphagia (five orally and ten via a nasogastric (NG) tube of which tip placement in the stomach was confirmed by chest X-ray).

**Table 1:** Baseline clinical characteristics of patients.

	Overall (n = 110)	15 mg QD (n = 59)	10 mg QD (n = 51)	P value
Women	37(34)	13(22)	24(47)	0.008
Age, y	74.6±9.4	68.8±7.4	81.4±6.6	<0.001
Congestive heart failure	13(12)	3(5)	10(20)	0.035
Hypertension	70(64)	36(61)	34(67)	0.558
Diabetes mellitus	29(26)	15(25)	14(27)	0.831
Index cerebrovascular events				0.949
Acute ischemic stroke	84(76)	46(78)	38(74)	
Acute TIA	8(7)	5(9)	3(6)	
Acute intracerebral hemorrhage	7(6)	2(3)	5(10)	
Chronic ischemic stroke	11(10)	6(10)	5(10)	
Prior vascular disease	11(10)	5(9)	6(12)	0.752
CHADS_2_	2(1–3)	1(1–2)	2(2–3)	0.001
CHA_2_DS_2_-VASc	3(2–4)	2(1–4)	4(3–5)	0.001
Weight	59.1±11.0	64.0±9.4	53.4±10.0	<0.001
NIHSS score on admission	4(2–14)	4(2–13)	5(1–15)	0.727
Concomitant use of antiplatelet agent	8(7)	4(7)	4(8)	0.831
Creatinine clearance (mL/min)	61.6±20.0	74.0±16.7	47.2±12.4	<0.001
30–49 mL/min	37(34)	0(0)	37(73)	<0.001
Liver dysfunction				
Child-Pugh grade B or C	0(0)	0(0)	0(0)	0.999
Tablet crushing	15(14)	5(9)	10(20)	0.103
Time from initiation of rivaroxaban to blood sampling, day	6(5–7)	6(5–7)	6(5–8)	0.625
Time from stroke/TIA onset to blood sampling, day*	12(8–15)(n = 99)	12(8–13)(n = 53)	12(9–15)(n = 46)	0.212

Data are numbers (%), means±SD, or medians (interquartile range). *Patients with chronic ischemic stroke are excluded. TIA, transient ischemic attack; NIHSS, National Institutes of Health Stroke Scale.

### Coagulation markers and estimated rivaroxaban concentration

The distribution of plasma coagulation markers is shown in Table 2. Among the three sampling points, 99 patients (90%) reached the highest estimated concentration of rivaroxaban at 4 h, while the other 11 (10%) reached it at 9 h. The baseline characteristics of these 11 patients did not differ from those of the remaining 99 patients.

**Table 2:** Coagulation markers and estimated rivaroxaban concentration.

	0 h	4 h	9 h
aPTT, sec	32(29–34)	43(37–48)	37(34–41)
PT, sec	12.8(12.1–13.7)	19.4(16.7–22.3)	16.3(14.5–18.2)
PT-INR	1.04(0.98–1.11)	1.56(1.34–1.80)	1.32(1.17–1.47)
Rivaroxaban concentration, ng/mL	11(5–22)	168(109–243)	65(44–103)

aPTT, activated partial thromboplastin time; PT, prothrombin time; INR, international normalized ratio.

**Figure 1:**
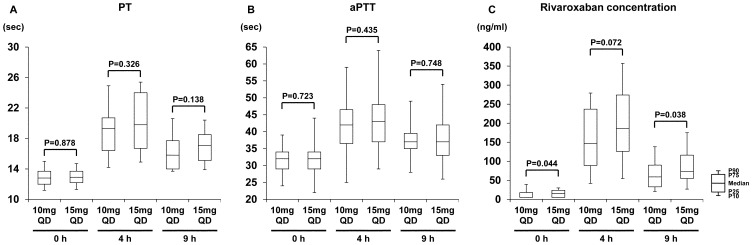
PT (A), aPTT (B), and rivaroxaban concentration (C_riv_) at 0 h, 4 h, and 9 h after administration.

Coagulation markers and C_riv_ at 0 h, 4 h, and 9 h after administration of two different dosages are shown in Figure 1. Between the two dosage groups, there were no significant differences in aPTT and PT at all sampling points. The 15 mg QD group demonstrated higher rivaroxaban concentrations at 0 h and 9 h than the 10 mg QD group [Figure 1], and the median C_riv_ was 27% higher at 4 h (186 ng/mL vs. 147 ng/mL) and 24% higher at 9 h (73 ng/mL vs. 59 ng/mL).

**Figure 2:**
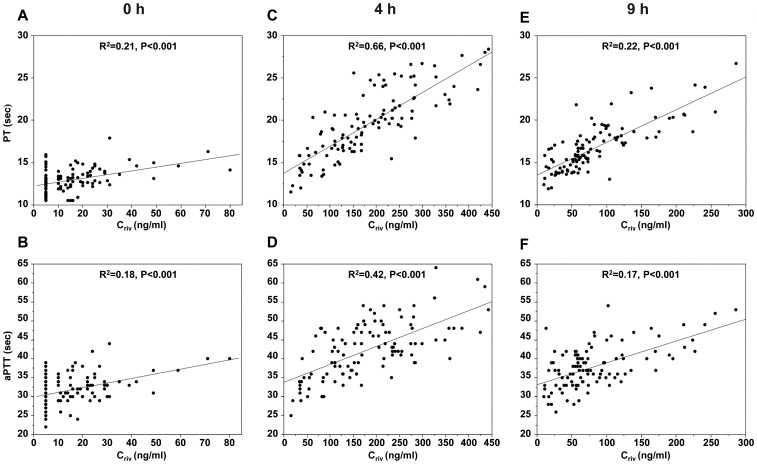
Correlations of estimated rivaroxaban concentration (C_riv_) with PT (sec) and aPTT (sec) at 0 h (A, B), 4 h (C, D), and 9 h (E, F).

Both PT and aPTT values were prolonged in a concentration-dependent manner, and they showed positive correlations with C_riv_ at 0 h, 4 h, and 9 h (Figure 2). The linearity of the relationship seen between PT and C_riv_ had a higher R^2^ value than that between aPTT and C_riv_.

**Figure 3:**
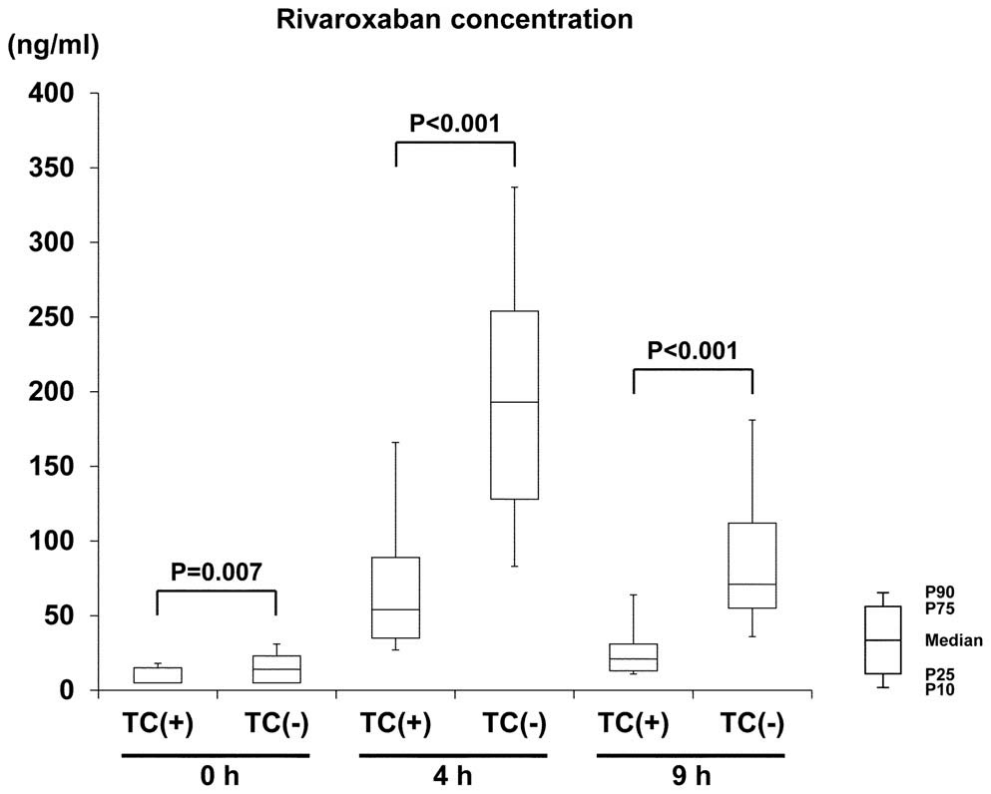
Comparison of rivaroxaban concentrations between groups with tablet crushing [TC (+)] and those without [TC (−)]. TC indicates tablet crushing.

In comparison to the 95 patients receiving regular tablets, the other 15 patients receiving crushed tablets showed lower rivaroxaban concentrations at all three time points [Figure 3]; the median C_riv_ was 72% lower at 4 h (54 ng/mL vs. 193 ng/mL, P<0.001) and 70% lower at 9 h (21 ng/mL vs. 71 ng/mL, P<0.001). C_riv_ did not differ between the ten patients receiving crushed tablets via an NG tube (median 46 ng/mL at 4 h) and the five patients receiving oral administration (median 69 ng/mL, P = 0.624). After exclusion of these 15 patients, the median C_riv_ at 4 h of the 95 patients receiving regular tablets was 193 ng/mL (206 ng/mL for 54 patients on 15 mg QD and 168 ng/mL for 41 patients on 10 mg QD). The median PT (15.8 sec vs. 20.0 sec at 4 h, P<0.001) and aPTT values (14.5 sec vs. 16.7 sec t 4 h, P<0.001) were also shorter in patients receiving crushed tablets than in the other patients.

In addition to tablet crushing (P<0.001), congestive heart failure (P = 0.073), diabetes mellitus (P = 0.005), NIHSS score on admission (P = 0.022), rivaroxaban dosage (P = 0.029), and time from stroke/TIA onset to blood sampling (P = 0.010) were identified as variables with P<0.20 by the preceding analysis for the linear mixed-effect model. Table 3 provides the adjusted estimates and 95% confidence intervals for the linear mixed-effect model. The results showed that diabetes mellitus (P = 0.029), time from stroke/TIA onset to blood sampling (P = 0.047), and tablet crushing (P<0.001) were significantly associated with C_riv_.

**Table 3:** Linear mixed-effect model to determine variables that influence rivaroxaban concentration.

Variable		Adjusted Estimate (95%CI)	P value
Congestive heart failure	No	Reference	0.482
	Yes	0.07 (−0.11 to 0.23)	
Diabetes mellitus	No	Reference	0.029
	Yes	0.13 (0.01 to 0.25)	
NIHSS score, per 1 point		−0.01(−0.02 to 0.01)	0.346
Rivaroxaban dosage	15 mg QD	Reference	0.146
	10 mg QD	−0.08 (−0.19 to 0.03)	
Tablet crushing	No	Reference	<0.001
	Yes	−0.43 (−0.60 to −0.26)	
Time from stroke/TIA onset to blood sampling, per day		−0.02 (−0.03 to 0.01)	0.047
Time points of blood sampling	0 h	Reference	
	4 h	1.13 (1.06 to 1.20)	<0.001
	9 h	0.29 (0.22 to 0.37)	<0.001

NIHSS, National Institutes of Health Stroke Scale; TIA, transient ischemic attack.

## Discussion

In the present study, the outcomes of conventional clotting tests and anti-factor Xa chromogenic assays in Japanese stroke patients taking rivaroxaban were evaluated to assess the anticoagulation intensity of rivaroxaban and explore its determinant factors. The anti-factor Xa chromogenic assay has showed acceptable accuracy and precision for quantitative measurements of rivaroxaban exposure, using rivaroxaban calibrators. The first major finding of this study was that C_riv_ at 4 h, indicating nearly peak concentration, was relatively low as compared with the maximum C_riv_ values in the ROCKET AF and J-ROCKET AF trials. The second major finding was that tablet crushing decreased anticoagulation intensity.

Since stroke patients are often aged and often have renal dysfunction [14],[15], lower dosages of NOACs tend to be chosen for such patients. In particular, special low dosages of rivaroxaban are recommended in Japan. Thus, we had a concern that C_riv_ in Japanese stroke patients was much lower than C_riv_ from global data. On the other hand, by transiently worsened renal function in the acute stroke setting, there was also a concern about accidental elevation of C_riv_. According to exposure simulations performed in patients included in the ROCKET AF and J-ROCKET AF trials, the maximum C_riv_ in Japanese patients with 15 mg QD (mean 249 ng/mL, J-ROCKET AF) was identical with that in non-Japanese patients with 20 mg QD (mean 249 ng/mL, ROCKET AF), and the maximum C_riv_ in Japanese with 10 mg QD (mean 168 ng/mL) was lower than that in non-Japanese with 15 mg QD (mean 229 ng/mL) [4]. These levels were still higher than the mean C_riv_ at 4 h in the present patients (197 ng/mL for 15 mg QD, 163 ng/mL for 10 mg QD). A reason for the large difference in C_riv_ was inclusion of patients receiving crushed tablets in the present study, since the mean C_riv_ at 4 h only in the patients receiving uncrushed tablets showed smaller differences from previous data (207 ng/mL for 15 mg QD, 188 ng/mL for 10 mg QD).

Previous studies demonstrated an 18% decrease in maximum C_riv_ for the crushed tablets suspended in water and administered via an NG tube followed by a liquid meal, compared to that after the whole tablet [10],[16], and a 29% decrease in AUC and a 56% decrease in maximum C_riv_ when the granulate was directly released into the proximal small intestine immediately followed by food. Thus, absorption of rivaroxaban seems to be dependent on the site of drug release in the gastrointestinal tract [10]. Indeed, the manufacturer recommends avoiding administration of rivaroxaban directly into the proximal small intestine (e.g., feeding tube) and illustrates the administration of crushed tablets via an NG tube or gastric feeding tube as a special option if patients are unable to swallow whole tablets. However, the present differences in C_riv_ between patients receiving crushed tablets and those receiving whole tablets were more divergent (72% at 4 h) than the above-mentioned results. Since rivaroxaban tablets are small, practically insoluble in water, and need to be crushed and suspended in water instead of a simple suspension method when administered via an NG tube [10], drug loss in the grinding, sifting, and packaging processes or drug remaining in the syringe and NG tube may occur.

Another possible reason for the low C_riv_ in the present patients was that the data were based on fixed time-point measurements, not on consecutive measurements to identify the peak level. The timing of blood sampling at 4 h in the present study was determined based on the previous finding noted in the Methods [10]. However, 10% of the present patients showed higher C_riv_ at 9 h than at 4 h, suggesting that the peak concentration time could be delayed in the clinical setting of acute stroke care, probably because the patients are old and often have renal dysfunction. Additionally, a previous phase-1 study displayed minor double peaks in rivaroxaban concentration after receiving crushed tablets via NG tubes; the first peak occurring around 45 minutes, and the second one between 4 and 6 h [16]. Our sampling timing at 4 h may be the nadir of biphasic peaks.

Although reduced CrCl is the only criterion for selecting a low dosage of rivaroxaban (10 mg QD) in Japan, 14 patients without a reduced CrCl were also given a low dosage based on the judgments of the physicians in charge because they had a history of bleeding or were very old. Such judgments appeared to contribute to the present low C_riv_ values. In addition, some patients might show higher serum creatinine levels in the acute stage of stroke than usual due to hypovolemia and potential damage by acute stroke. CrCl in such patients might return to higher levels within several days; that might be another cause of the present low C_riv_ values.

The present study showed a linear relationship between PT and C_riv_, as was also shown in the J-ROCKET AF and ROCKET AF trials [4],[17]. The present study also showed a linear relationship between aPTT and C_riv_, although the R^2^ level was lower than that of PT, and most earlier publications showed that aPTT is less sensitive than PT for rivaroxaban exposure assessment [18],[19]. However, the aPTT and PT results should be carefully interpreted because their sensitivities depend on the reagents.

The unique point of the present study was that most of the studied patients were enrolled into the study soon after onset of stroke or TIA; such acute patients were excluded from the major clinical trials [5],[6],[7],[8],[9] and have been infrequently studied after the approval of clinical use of NOACs. Although the optimal timing for initiation of NOACs has not been established, none of 412 patients who began to take NOACs in acute stage of ischemic stroke/TIA did not develop intracranial hemorrhage during acute hospitalization in our ongoing multicenter observational SAMURAI-NVAF study (Toyoda K, et al: unpublished data). The limitations of the present study included a relatively small sample size and the poor estimation accuracy of the anti-Xa chromogenic assay for C_riv_ when the concentration is low, as well as the fixed-point measurement of anticoagulation intensity.

In conclusion, this is the first study of Japanese stroke patients examining the anticoagulation intensity of rivaroxaban. The impressive finding was that tablet crushing, required for dysphagic patients, who are common in stroke medicine, decreased rivaroxaban concentration. Thus, tablet crushing should be carefully considered. At the least, patients with CrCl ≧50 mL/min should not be given a lower dosage (10 mg QD) when they need tablet crushing.
